# Heterogeneous temporal representation for diabetic blood glucose prediction

**DOI:** 10.3389/fphys.2023.1225638

**Published:** 2023-07-17

**Authors:** Yaohui Huang, Zhikai Ni, Zhenkun Lu, Xinqi He, Jinbo Hu, Boxuan Li, Houguan Ya, Yunxian Shi

**Affiliations:** ^1^ College of Electronic Information, Guangxi Minzu University, Nanning, China; ^2^ Laboratory of Intelligent Information Processing and Intelligent Medical, Guangxi Minzu University, Nanning, China; ^3^ Department of Electronic Science, Xiamen University, Xiamen, China

**Keywords:** continuous glucose monitoring, diabetes mellitus, time series, prediction, graph neural network, deep neural network

## Abstract

**Background and aims:** Blood glucose prediction (BGP) has increasingly been adopted for personalized monitoring of blood glucose levels in diabetic patients, providing valuable support for physicians in diagnosis and treatment planning. Despite the remarkable success achieved, applying BGP in multi-patient scenarios remains problematic, largely due to the inherent heterogeneity and uncertain nature of continuous glucose monitoring (CGM) data obtained from diverse patient profiles.

**Methodology:** This study proposes the first graph-based Heterogeneous Temporal Representation (HETER) network for multi-patient Blood Glucose Prediction (BGP). Specifically, HETER employs a flexible subsequence repetition method (SSR) to align the heterogeneous input samples, in contrast to the traditional padding or truncation methods. Then, the relationships between multiple samples are constructed as a graph and learned by HETER to capture global temporal characteristics. Moreover, to address the limitations of conventional graph neural networks in capturing local temporal dependencies and providing linear representations, HETER incorporates both a temporally-enhanced mechanism and a linear residual fusion into its architecture.

**Results:** Comprehensive experiments were conducted to validate the proposed method using real-world data from 112 patients in two hospitals, comparing it with five well-known baseline methods. The experimental results verify the robustness and accuracy of the proposed HETER, which achieves the maximal improvement of 31.42%, 27.18%, and 34.85% in terms of MAE, MAPE, and RMSE, respectively, over the second-best comparable method.

**Discussions:** HETER integrates global and local temporal information from multi-patient samples to alleviate the impact of heterogeneity and uncertainty. This method can also be extended to other clinical tasks, thereby facilitating efficient and accurate capture of crucial pattern information in structured medical data.

## 1 Introduction

Diabetes mellitus is a chronic metabolic disorder that afflicts over 463 million adultsworldwide, posing significant health risks and economic burdens (Bellary et al., 2021; Flood et al., 2021). Hyperglycemia is the hallmark symptom of diabetes mellitus and can lead to serious complications such as cardiovascular disease, blindness, and heart failure (Cole and Florez, 2020). Blood glucose levels are crucial health indicators for patients with diabetes (Leelarathna et al., 2022). Continuous Glucose Monitoring (CGM) is a medical device that enables patients to regularly track their blood glucose levels (Battelino et al., 2022; Jin et al., 2022; Johnson et al., 2021). CGM data can provide valuable insights into the dynamics of glucose metabolism, which can assist in optimizing the treatment strategies for both patients and clinicians (Visser et al., 2023). However, CGM data inherently exhibit heterogeneity and uncertainty, as they vary depending on the type of diabetes, duration of monitoring, and individual characteristics of each patient (Martens et al., 2021; Elbalshy et al., 2022). Therefore, developing effective blood glucose prediction (BGP) methods based on CGM data from multiple patients remains challenging.

Data-driven methods have received significant attention from researchers in recent years (Qiao et al., 2023; Wang et al., 2023), which learn potential patterns from historical observations and make inferences for the future. These methods are widely applied in healthcare, including disease prediction and diagnosis (Zhang P. et al., 2022; Huang et al., 2022; Cai et al., 2019), treatment decision support (Wang et al., 2022), medical image analysis Cai et al. (2020), and health monitoring (such as CGM, blood pressure monitoring, body temperature monitoring, and others) (Wang et al., 2021; Zhang H. et al. 2022). Previous studies on CGM have proposed a variety of machine learning and deep learning models for predicting blood glucose levels (Wadghiri et al., 2022; Zhang P. et al., 2022; Ye et al., 2022) employed the swallow machine learning method, such as random forester (RF) and support vector machine (SVM), to achieve efficient glucose monitoring. Zhu et al. (2023) proposed an attention-based recurrent neural network to BGP for type 1 diabetes mellitus (T1DM) patients. Both Rabby et al. (2021) and Yang et al. (2022) have designed a hybrid long short-term memory network (LSTM) and demonstrated promising performance for T1DM patients. Aliberti et al. (2019) attempted to use the glucose signal by multiple patients to infer a new patient glucose-level through RNN, LSTM, and auto-regressive model; Deng et al. (2021) presented an improved generative adversarial network to capture the temporal patterns from type 2 diabetes mellitus (T2DM) patients. Similar works also can be found from (Xie and Wang, 2020; Nemat et al., 2022; Lee et al., 2023; Mhaskar et al., 2017). Nevertheless, the preponderance of these approaches is concentrated on personalized Blood Glucose Prediction (BGP) and lacks the capacity to be generalized to multi-patient forecasting scenarios.

There are three major challenges that limit the prediction accuracy. 1) *Heterogeneity*: Monitoring durations vary among patients, attributable to individual differences in diagnosis, treatment, and lifestyle choices. This variation undermines the effectiveness of a one-size-fits-all approach to multi-patient blood glucose prediction (Espinoza et al., 2023; Hollander and Roep, 2022), thereby exerting a significant impact on the performance of predictive models in multi-patient monitoring scenarios. 2) *Uncertainty*: The uncertainty regarding the specific type of diabetes (T1DM or T2DM) can impact the accuracy of CGM predictions in multi-patient scenarios, given the significant potential divergence in the progression of these two types Eizirik et al. (2020). Moreover, coordinating continuous glucose monitoring (CGM) data from multi-patients introduces additional uncertainties, especially during overlapping, partially overlapping, or non-overlapping periods. These discrepancies can influence data consistency and model reliability Karges et al. (2023). 3) *Correlation*: The prediction model must account for potential correlations that exist across both temporal and spatial dimensions among the samples or periods Zhang P. et al. (2022). These correlations could stem from shared environmental factors, similar treatment plans, or communal lifestyle habits. Neglecting these correlations can impact the accuracy of predictions Zale and Mathioudakis (2022).

To address the aforementioned challenges, we propose a novel heterogeneous representation learning model for diabetic blood glucose prediction, called HETER (heterogeneous temporal representation network). This model can be applied to multiple patients with various monitoring durations and different types of diabetes. Compared to conventional BGP models, our proposed model structure employs the subsequence repetition (SSR) method to process the heterogeneous CGM data. This approach avoids the information loss and meaningless incorporation often experienced with traditional truncation and padding methods Ahmed et al. (2023). To discover the potential correlation in both spatial and temporal dimensions within heterogeneous and uncertain CGM data, we employ a dynamic time-warping (DTW) approach. This approach measures the similarity among diverse samples and reconstructs the CGM data input from multiple patients into a directed graph. Subsequently, a graph learning component grounded on graph convolutional networks (GCNs) is devised to capture the spatial characteristics embedded in the temporal information of the graph. These characteristics are frequently neglected by traditional recurrent learning-based approaches. This is the first work to leverage a graph neural network for multi-patient BGP to the best of our knowledge. In addition, HETER integrates a combination of attention and recurrent learning mechanisms to enhance the discrimination of distinct trends and patient types, thereby improving the robustness and generalizability for heterogeneous multi-patient BGP.

The major contributions can be summarized below.

1) The use of the SSR method for aligning heterogeneous CGM data is first applied in multi-patient blood glucose prediction. This approach presents a promising solution for processing heterogeneous medical time series data without the need for data truncation or interpolation.
2) A novel graph-based representation learning method is proposed to extract crucial temporal information from multiple CGM time series subsequences. To address the limitations of graph neural networks in capturing continuous temporal dynamics, we incorporate a representation enhancement module, further mining pattern information across both temporal and spatial dimensions.
3) The proposed HETER has been comprehensively evaluated by comparing it with five well-established prediction methods using a real-world heterogeneous CGM dataset. The results demonstrate the superiority of HETER in blood glucose prediction.

The rest of this paper is organized as follows: Section 2 introduces the materials and methods used in this study, including the dataset, problem definition, and methodology. Experimental results and analysis are presented in Section 3. Section 4 concludes the study by discussing its implications, limitations, and future research directions.

## 2 Materials and methods

### 2.1 Datasets

**FIGURE 1:**
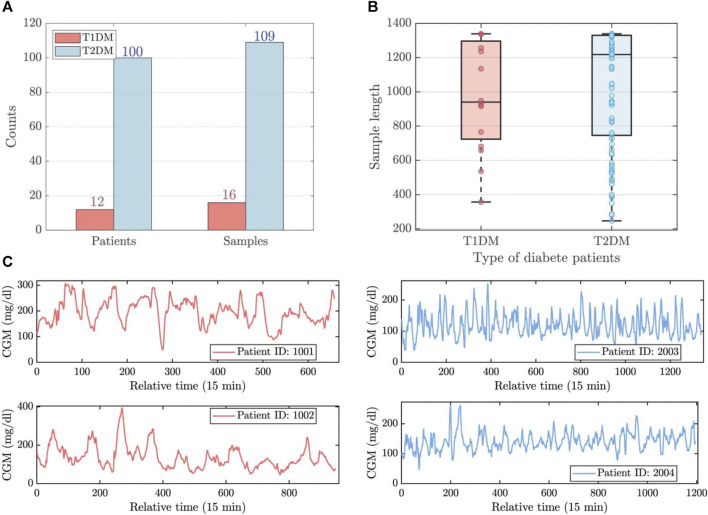
The brief visualization of experimental data. **(A)** The number of patients and samples for T1DM and T2DM. **(B)** The length distribution of T1DM and T2DM samples. **(C)** The time series observations of T1DM (left) and T2DM (right) samples.

The patients of experimental data were recruited from Shanghai East Hospital (from September 2019 to March 2021), and Shanghai Fourth Peopleś Hospital (from June 2021 to November 2021) in Shanghai, China Zhao et al. (2023). The concise overview of the dataset is visually represented in Figures 1A,B. This dataset comprises 125 CGM records from 112 patients, including 12 individuals with Type 1 Diabetes Mellitus (T1DM) and 100 individuals with Type 2 Diabetes Mellitus (T2DM). Patient sample lengths are inconsistent, as shown in Figure 1C. The maximum, mean, and minimum lengths of the T1DM samples stand at 1,339, 981, and 357, respectively, while for the T2DM samples, these values are 1,339, 1,031, and 247. The resolution of the data is 15 min.

Given these diabetes samples, three types of uncertainties arise: 1) sample length uncertainty: the lengths of each sample are inconsistent. 2) disease type uncertainty: this dataset comprises data from two types of patients, and predictions need to be made simultaneously for both types. 3) sample correlation uncertainty: potential temporal pattern associations may exist asynchronously among the various samples. These inherent uncertainties inevitably limit the accuracy of prior prediction methods, particularly when applied to the simultaneous tracing of multiple patients. In this study, we aim to build the complex relationship between subsequences for accurate prediction, utilizing a combination of graph neural networks, attention mechanisms, and recurrent learning components.

### 2.2 Problem definition

This section covers preliminaries of heterogeneous time series (HTS) and describes the problem of HTS prediction problems. The frequently used symbols are listed inSupplementary Table S1.

The continuous glucose monitoring data, collected from diverse patients at consistent intervals, demonstrates variation in relation to both monitoring durations and the specific initiation and termination timestamps. These data can be called the heterogeneous time series (HTS) in clinical glucose monitoring Duchêne et al. (2007); Zhang et al. (2017), denoted as $D\in R^{N\times L}$ , where *N* and *L* represent the number of samples and the preset length of each time series, respectively. The determination of length *L* is contingent upon the particular data processing approach employed, such as padding or truncation.

In this study, we consider both single-step and multi-step blood glucose prediction problems within the framework of HTS, which can be formulated as follows:

$$\left\{{\hat{D}}_{t+1},\ldots ,{\hat{D}}_{t+s}\right\}\leftarrow F\left(D_{t-T},\ldots ,D_{t}\right),$$ (1)

where F(⋅) denotes the prediction model, *t* represents the beginning time step, and *s* is the prediction steps. *T* indicates the window size, which means the length of the observational window of historical data we consider when making predictions. It determines the number of input features utilized for our forecasting endeavors. $\hat{D}$ represents the predictions.

### 2.3 Methodology

#### 2.3.1 Overview

**FIGURE 2:**
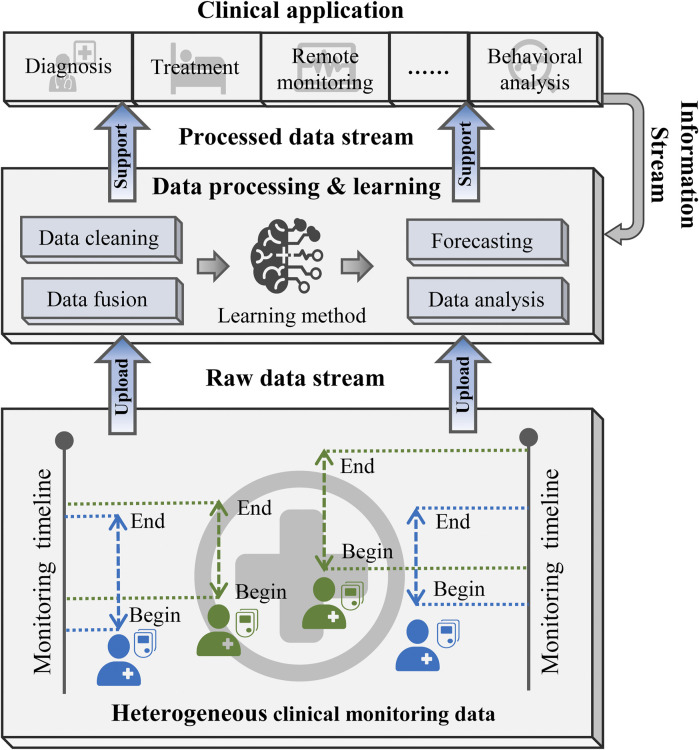
The workflow of clinical diabetic blood glucose prediction.

Figure 2 illustrates the general framework of the diabetic blood glucose prediction methods for multiple patients in clinical applications. First, heterogeneous CGM signals are aggregated into a raw data stream and delivered into the learning structure. Then, the learning structure employs data cleaning and fusion techniques to reorganize the input data. The processed data are then fed into learning methods for predictive modeling or other analytical purposes. Finally, the predictive outcomes and analytical findings can support clinical diagnosis, treatment strategies, and the implementation of precision medicine.

#### 2.3.2 Data alignment

The heterogeneity and uncertainty are inherent in CGM data samples present a significant challenge to traditional personalized prediction models, impeding their generalization to multi-patient tasks Capobianco (2017). Consequently, the alignment of sample lengths becomes a necessity. Truncation (TRA) and padding (PAD) are two prevalent techniques employed to ensure uniformity in sample lengths Chen et al. (2020); Hammad et al. (2021). Truncation, which involves reducing data to a pre-determined length, inevitably leads to the loss of potentially crucial information. On the other hand, padding, which necessitates adding artificial data to attain a specified length, risks incorporating extraneous and meaningless data, potentially reducing the accuracy of predictions.

**FIGURE 3:**
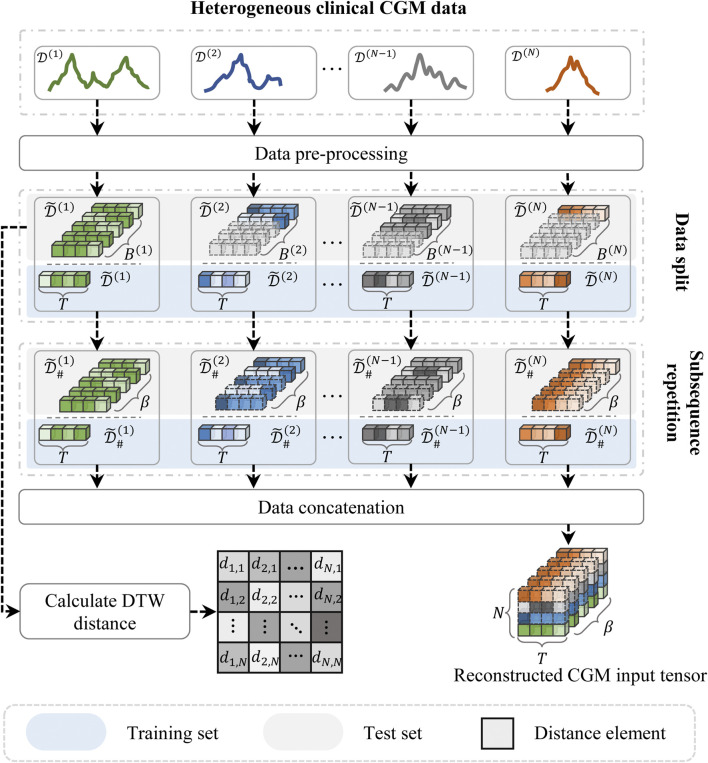
The schematic illustration of subsequence repetition (SSR) method.

To address these issues, we proposed a subsequence repetition (SSR) method to align and reconstruct the heterogeneous CGM data, as illustrated in Figure 3. First, the input data are individually normalized to scalar values using the min-max normalization technique in order to minimize the impact of magnitude differences. The process of normalization and de-normalization is formulated as follows:

$${\tilde{D}}^{\left(n\right)}=\frac{D^{\left(n\right)}-\min\left(D^{\left(n\right)}\right)}{\max\left(D^{\left(n\right)}\right)-\min\left(D^{\left(n\right)}\right)},\;$$ (2)

$$D^{\left(n\right)}={\tilde{D}}^{\left(n\right)}\left(\max\left(D^{\left(n\right)}\right)-\min\left(D^{\left(n\right)}\right)\right)+\min\left(D^{\left(n\right)}\right),$$ (3)

where ${\tilde{D}}^{\left(n\right)}$ and *D* ^(*n*)^ denote the *n*th normalized data and input data, respectively. max (⋅) is the maximal values of *D* ^(*n*)^, and min (⋅) represents the minimal values.

Subsequently, the normalized time series data are segmented into fixed-length subsequences, each representing a continuous time period consisting of *T* steps (i.e., window size). The generations after segmentation can be denoted as $\tilde{D}=\left\{{\tilde{D}}_{\#}^{\left(1\right)}\in R^{B^{\left(1\right)}\times T},\ldots ,{\tilde{D}}_{\#}^{\left(N\right)}\in R^{B^{\left(N\right)}\times T}\right\}$ . ${\tilde{D}}^{\text{train}}$ and ${\tilde{D}}^{\text{test}}$ are used to denote the training set and test set, respectively, after data splitting. In the test set, we maintain a consistent number of segments across all samples. Nevertheless, due to the uncertainty of sample length, the number of split segments within the training set remains inconsistent across the samples. Hence, we employ SSR to achieve alignment of segments in the training set, which can be obtained by:

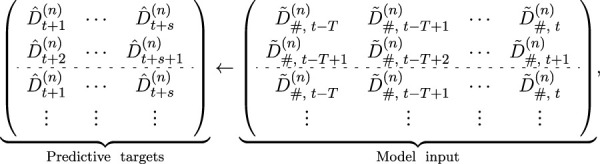

(4)
where *s* denotes the prediction steps, and *T* is the window size. The known subsequence segments are sequentially replicated until the total segment count equals *β*, where *β* represents the maximum number of segments within the training set. The reconstructed training data can be denoted as ${\tilde{D}}_{\#}^{\text{train}}=\left\{X_{\#}^{\text{train}},Y_{\#}^{\text{train}}\right\}$ and the test data is ${\tilde{D}}_{\#}^{\text{test}}=\left\{X_{\#}^{\text{test}},Y_{\#}^{\text{test}}\right\}$ .

The SSR method aligns the heterogeneous input data by utilizing the known data samples to fill the null subsequences. This method might increase redundant subsequence information, but it avoids losing the original temporal dependencies of the time series data. Both padding and truncation can easily affect the distribution of raw data.

#### 2.3.3 Graph construction

**FIGURE 4:**
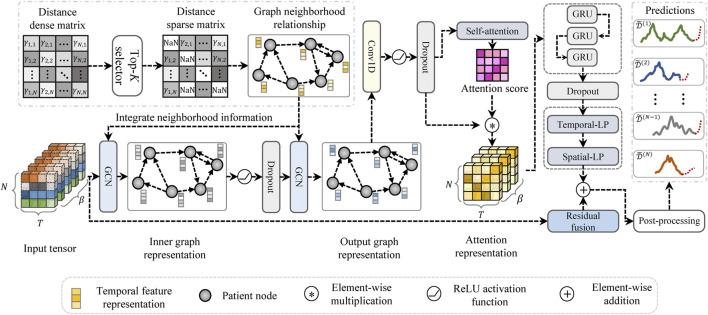
The architecture of the proposed heterogeneous temporal representation (HETER) network.

The model architecture of the proposed HETER is illustrated in Figure 4. In HETER, we initially calculate the distance between each heterogeneous CGM record in the training set, then construct a sparse relation graph (SRGraph) based on the obtained distance matrix. SRGraph is capable of capturing the intricate relationships among patients by arranging the input data in a graph structure. This is particularly crucial when considering the heterogeneity of individuals’ blood glucose levels influenced by varying lifestyle choices, treatment regimens, and genetic factors. Additionally, SRGraph excels in uncovering latent correlations between samples or periods across both spatial and temporal dimensions. This makes SRGraph particularly suitable for multiple patients’ BGP, where correlations may exist but are not explicitly evident in the data.

Due to the inconsistent sample length among patients, the distance cannot be calculated on a point-to-point basis. Consequently, we employ the dynamic time-warping (DTW) approach to align the sample lengths of the raw input data Li et al. (2020). This method is a widely used approach for measuring similarity between time series Li (2021). It aims to minimize the distance between two time series while aligning their sample lengths. The process can be expressed as follows:

$$\begin{array}{cc} \gamma_{i,j} & =distance\left(D^{\left(i\right)},D^{\left(j\right)}\right)\\ & =\underset{\Lambda }{\min}\underset{\left(s^{1},s^{2}\right)\in \Lambda }{\sum }distance\left(d_{i,s^{1}},d_{j,s^{2}}\right), \end{array}$$ (5)

where *γ*_*i*,*j*_ represents the calculated distance between samples *i* and *j*. Λ = [*λ* ^1^, *…* , *λ* ^*κ*^] denotes the optimal alignment path between two samples. The alignment path *λ* = (*s*_1_, *s*_2_) must satisfy 1 ≤ *s*_1_ ≤ *L*_*i*_ and 1 ≤ *s*_2_ ≤ *L*_*j*_, where *s*_1_ and *s*_2_ represent the time points of the samples, and *L*_*i*_ and *L*_*j*_ denote the sample lengths of *D* ^(*i*)^ and *D* ^(*j*)^, respectively. The path begins at *λ* ^1^ = (0, 0) and ends at *λ* ^*κ*^ = (*L*_*i*−1_, *L*_*j*−1_). Both *d*_*i*_ ∈ *D* ^(*i*)^ and *d*_*j*_ ∈ *D* ^(*j*)^ represent observations from the CGM record. The distance matrix for the input time series can be expressed as Γ = [*γ*_1,2_, *…* , *γ*_*N*,*N*−1_].

Then, the formal definition of the proposed SRGraph can be described below.

*Definition 1 (SRGraph).* The SRGraph is formulated as G=(V,E) , where $V\in R^{N}$ is the set of nodes (i.e., CGM samples), and $E\in R^{2\times N\times K}$ is the set of edges (i.e., the relationships between CGM samples). *N* is the sample size and is equal to the number of nodes in the graph. *K* is a hyperparameter that denotes the number of items we aim to retrieve in the top positions of the distance ranking.

*Definition 2 (Node Neighborhood).* Let *v*_*i*_, *v*_*j*_ ∈ *V* denote nodes in the graph, and $E_{i,j}=\left(v_{i},v_{j}\right)$ to denote an directed edge pointing from *v*_*i*_ to *v*_*j*_. The neighborhood of a node *v*_*i*_ is represented as $N\left(v_{i}\right)=\left\{v_{j}\in V|\left(v_{i},v_{j}\right)\in E\right\}$ .

*Definition 3 (Adjacency Matrix).* The adjacency matrix is a mathematical representation of a graph. This study sorts the distances and selects the top-*K* minimal distances for each sample to construct the adjacency matrix. This matrix is denoted as $A\in R^{N\times K}$ . If $\left(v_{i},v_{j}\right)\in E$ , then *A*_*i*,*j*_ > 0, and if $\left(v_{i},v_{j}\right)\notin E$ , then *A*_*i*,*j*_ = 0.

In general, CGM samples are treated as nodes in the graphs. The edges of the graph are constructed based on the distance relationships between these samples. With the information about nodes and edges, we generate the graph adjacency matrix, which is subsequently learned by the graph structure learning module.

#### 2.3.4 Graph learning module

The proposed graph learning module intends to integrate the information of a node with that of its neighboring nodes to capture the temporal dependencies in a graph. Two stacked graph convolutional networks (GCNs) Zhang et al. (2019) are employed in the proposed HETER to embed the information of the SAGraph. This can be expressed as follows:

$$\begin{array}{cc} R_{g}^{\left(1\right)} & =W_{g}^{\left(1\right)}⋆X_{\#}\\ & ={\tilde{M}}^{-\frac{1}{2}}\tilde{A}{\tilde{M}}^{-\frac{1}{2}}X_{\#}W_{g}^{\left(1\right)}+b_{g}, \end{array}\;$$ (6)

$$R_{g}^{\left(2\right)}=W_{g}^{\left(2\right)}⋆Dropout\left(\varphi \left(R_{g}^{\left(1\right)}\right),p_{g}\right),$$ (7)

where symbol ⋆ indicates the graph convolution operation. $X_{\#}$ is the input for proposed model. $\tilde{M}\in R^{T\times T}$ denotes the diagonal matrix. The element ${\tilde{M}}_{i,i}$ represents the degree of node *i*, corresponding to the number of edges connected to node *i*. $\tilde{A}=A+I_{u}\in R^{N\times \left(K+1\right)}$ denotes an adjacency matrix with self-loops. ***A*** is the adjacency matrix of SAGraph and ***I***_*u*_ represents a unit matrix. $R_{g}^{\left(1\right)}$ is the embedding graph representation. $R_{g}^{\left(2\right)}$ is the output graph representation. *p*_*g*_ is dropout probability. *φ*(⋅) is rectified linear unit (ReLU) activation. ***W***_*g*_ represents the learnable weighting matrix. ***b***_*g*_ is the learnable bias.

#### 2.3.5 Representation enhancement module

The potential topological characteristics between patients are extracted by the graph learning module Wu et al. (2020). The representation enhancement module is designed to exploit further the key information in both the temporal and spatial dimensions.

First, a convolution component is employed to filter out the useless information in spatial dimensions, the Dropout technique also be integrated to prevent overfitting:

$$R_{co}=Dropout\left(\varphi \left(W_{co}*R_{g}^{\left(2\right)}\right),p_{co}\right),$$ (8)

where ***W***_*co*_ is the learnable convolution kernel, $R_{co}$ represents the output from the convolution component, and * denotes the traditional convolution operation. *p*_*c*_ is the dropout probability for the convolution component.

A limitation of graph convolutional networks is their inability to capture the continuous dynamical patterns from input CGM data. This is because the information propagation of graph convolutional networks occurs merely through nodes, ignoring the continuity of temporal features within nodes. To address this issue, temporal attention (TA) and a recurrent learning component are incorporated into the proposed HETER. Temporal attention is applied to highlight the key time steps in a period:

$$S_{ta}=softmax\left(W_{ta}^{\left(1\right)}\left(\varphi \left(W_{ta}^{\left(2\right)}R_{c}+b_{ta}^{\left(2\right)}\right)\right)+b_{ta}^{\left(1\right)}\right),$$ (9)

where $S_{ta}$ denotes the output attention score of TA. ***W***_*ta*_ and ***b***_*ta*_ are the learnable weighting matrix and bias, respectively.

HETER utilizes the attention score to reweight the output of the convolution component, which is then delivered to the gated recurrent unit (GRU)-based recurrent learning component to further extract the temporal dynamics. The information propagation processing in the recurrent learning component can be formulated as:

$$r_{t}=\sigma \left(W_{r}\left[h_{t-1};R_{co\;}{⊙\;S}_{ta}\right]+b_{r}\right),\;$$ (10)

$$u_{t}=\sigma \left(W_{u}\left[h_{t-1};R_{co\;}{⊙\;S}_{ta}\right]+b_{u}\right),\;$$ (11)

$$c_{t}=\sigma \left(W_{c}\left[r_{t}⊙h_{t-1};R_{co\;}{⊙\;S}_{ta}\right]+b_{c}\right),$$ (12)

$$h_{t}=\left(1-u_{t}\right)⊙h_{t-1}+u_{t}⊙c_{t},\;$$ (13)

where ***h***_*t*_ is the hidden state at *t* steps of GRU unit. ***u***_*t*_, ***r***_*t*_, ***c***_*t*_ denotes the update gate, reset gate, and candidate hidden state of recurrent learning component, respectively. *σ*(⋅) indicates the sigmoid activation function. [;] represents the concatenation operation, and ⊙ is the element-wise multiplication operation.

#### 2.3.6 Residual fusion and prediction

The output layer of the proposed HETER method consists of two multilayer perceptron networks, which are designed to encode the result into the desired output shape. To avoid the issue of vanishing or exploding gradients induced by excessive nonlinearity, the model’s linear input representation is added to the product of the output layer to yield the final prediction results:

$$R_{o}=SMLP\left(TMLP\left(Dropout\left(h_{t},p_{o}\right)\right)\right),$$ (14)

$${\hat{O}}_{\#}=R_{o}⊕\left(W_{o}X_{\#}+b_{o}\right),\;$$ (15)

where $R_{o}$ is the output from the generation layer, and ${\hat{O}}_{\#}$ represents the predictive values. *p*_*o*_ is the dropout probability, and ⊕ denotes the element-wise addition operation. ***W***_*o*_ and ***b***_*o*_ are the weight matrix and bias, respectively, used to generate the linear representation of the model input. The final results ${\hat{Y}}_{\#}$ are obtained after de-normalization by Eq. (3). Here, SMLP (⋅) and TMLP (⋅) correspond to Spatial-LP and Temporal-LP, respectively.

## 3 Results and discussion

### 3.1 Experimental setup

#### 3.1.1 Baseline methods

To verify the effectiveness of the proposed HETER, we select the following five methods as baselines for comparison. These methods have already achieved promising performance in personal diabetic blood glucose prediction or universal time series tasks.

1) **Vanilla LSTM** Rabby et al. (2021) is a fundamental long short-term memory (LSTM) model that utilizes its internal gating mechanisms to capture temporal dynamics.
2) **BiLSTM** Nemat et al. (2022) is a bi-directional recurrent neural network model that can capture both past and future temporal dynamics simultaneously.
3) **Transformer** Lee et al. (2023) exhibits superiority in capturing global dependencies through its self-attention mechanism.
4) **TPA-LSTM** Shih et al. (2019) incorporates attention mechanism and recurrent neural networks for processing time series data.
5) **LSTNet** Lai et al. (2018) is an improved convolutional and recurrent architecture that is capable of capturing long-term dependencies and periodic patterns.

#### 3.1.2 Performance criteria

We employ the mean absolute error (MAE), mean absolute percentage error (MAPE), and root mean square error (RMSE) to provide a comprehensive evaluation the performance of the predictive model. The MAE provides a straightforward metric of average error magnitude. The MAPE offers a scale-independent error measure in percentage terms, which is critical given the varying blood glucose levels across different contexts. The RMSE, emphasizing larger errors, assumes importance in our study, particularly considering the potential health consequences of substantial deviations in blood glucose levels. Moreover, these criteria are frequently adopted metrics for evaluating the accuracy of blood glucose predictions Xie and Wang (2020); Yang et al. (2023), which can be expressed as follows.

1) Mean absolute error

$$MAE=\mu \left(\sum_{\left(i,t\right)\in \Omega_{\text{test}}}\left|Y_{\#}^{i,t}-{\hat{Y}}_{\#}^{i,t}\right|\right),$$ (16)

2) Mean absolute percentage error

$$MAPE=\mu \left(\sum_{\left(i,t\right)\in \Omega_{\text{test}}}\left|\frac{Y_{\#}^{i,t}-{\hat{Y}}_{\#}^{i,t}}{Y_{\#}^{i,t}}\right|\right),$$ (17)

3) Root mean square error

$$RMSE=\sqrt{\mu \left(\sum_{\left(i,t\right)\in \Omega_{\text{test}}}{\left(Y_{\#}^{i,t}-{\hat{Y}}_{\#}^{i,t}\right)}^{2}\right)},$$ (18)

where $Y_{\#}^{i,t}$ and ${\hat{Y}}_{\#}^{i,t}$ denote the actual and predictive values at time step *t* of the *i* sample, respectively. *Ω*_test_ represents the test set. *μ*(⋅) denotes the operation of computing the mean values. The lower the values of MAE, MAPE, and RMSE, the better the prediction model’s performance.

#### 3.1.3 Model configurations

The experiments use the most recent 36 h (144 data points) of data for testing, while the remaining data were allocated for training. All experiments were conducted five times to improve the reliability of the results. The optimal hyperparameters were determined using the grid search method. The models were trained using the Adam optimization algorithm Kingma and Ba (2015), with mean squared error (MSE) employed as the loss function. The training epochs and learning rate are adjusted to approach their optimal states for each method. Supplementary Table S2 provides the detailed settings for the hyperparameters of each method. We implement the models using Porch (v.1.12.1) Paszke et al. (2019). The experiments for baseline methods and the proposed method were carried out on a server equipped with Intel(R) Xeon(R) Gold 6226R CPU (2.90 GHz) with 128G memory and were accelerated by two NVIDIA RTX A6000 GPUs.

### 3.2 Performance comparison

The experimental results comparing the performance of the baseline model and HETER across three prediction horizons *H* are displayed in Table 1. HETER outperforms comparable methods in terms of MAE, MAPE, and RMSE. We also observe that HETER, utilizing the SSR processing method, exhibits superior performance in most scenarios, particularly when the prediction horizon is established at 15. The TPA-LSTM with truncation (TRA) ranks second in most cases, and LSTNet shows the second-best performance in terms of RMSE when *H* is set to 30 and 60. This suggests the effectiveness of recurrent learning units. However, the inferior performance of Vanilla LSTM and BiLSTM, which consistently rank lower than other comparable methods, indicates that the output representations from recurrent units need additional refinement via other components, such as attention mechanisms or convolution layers. The proposed HETER improves MAE, MAPE, and RMSE by 31.42%, 27.18%, and 34.85%, over the second-best method when *H* = 15. When *H* is set to 30 and 60, the performance improvements over the second-best methods are 19.67% and 15.43% for MAE, 18.66% and 14.62% for MAPE, and 20.67% and 15.43% for RMSE, respectively.

**TABLE 1:** The performance comparison in three metrics and three horizons on CGM observations. The best result for each metric is highlighted in bold.

*H*	Metrics	Vanilla LSTM		BiLSTM		Transformer		TPA-LSTM		LSTNet		HETER (ours)		
(min)	(mg/dL)	TRA	PAD	TRA	PAD	TRA	PAD	TRA	PAD	TRA	PAD	TRA	PAD	SSR
15	MAE	30.724	34.959	30.571	32.566	28.537	31.569	5.924	7.769	6.050	6.542	4.193	4.372	**4.063**
	MAPE	0.243	0.270	0.244	0.259	0.232	0.250	0.045	0.059	0.047	0.051	0.034	0.034	**0.033**
	RMSE	42.123	48.664	41.918	44.463	38.622	43.650	8.754	11.323	8.781	9.295	5.872	6.329	**5.703**
30	MAE	30.688	35.631	30.599	32.982	29.560	32.295	8.415	10.460	8.368	9.702	7.575	6.784	**6.722**
	MAPE	0.245	0.274	0.243	0.265	0.244	0.257	0.064	0.082	0.064	0.076	0.060	**0.052**	0.054
	RMSE	42.024	49.546	42.045	44.750	40.019	44.389	12.943	15.319	12.751	13.937	11.352	10.531	**10.115**
60	MAE	30.858	36.211	30.586	33.129	29.733	34.604	12.830	14.399	12.976	14.941	11.233	**10.850**	10.980
	MAPE	0.237	0.282	0.243	0.265	0.237	0.271	0.097	0.111	0.103	0.114	0.090	**0.082**	0.089
	RMSE	42.828	50.319	42.098	44.836	40.416	47.836	19.856	21.422	19.618	21.474	16.971	17.200	**16.591**

When the prediction horizon *H* is set to 30 and 60, HETER with SSR exhibits competitive RMSE performance in comparison to HETER with padding (PAD), but it performs inferior in terms of MAPE. This can be attributed to the nature of RMSE and MAPE. Specifically, RMSE assigns greater weight to high-magnitude data points (such as sharp increases and decreases), while MAPE focuses more on the relative error between predicted and actual values. In other words, HETER with PAD generally performs well, while HETER with SSR achieves superior accuracy during periods of high volatility. Moreover, these results also demonstrate the efficacy of the proposed HETER in filtering out meaningless information.

### 3.3 Prediction analysis

**FIGURE 5:**
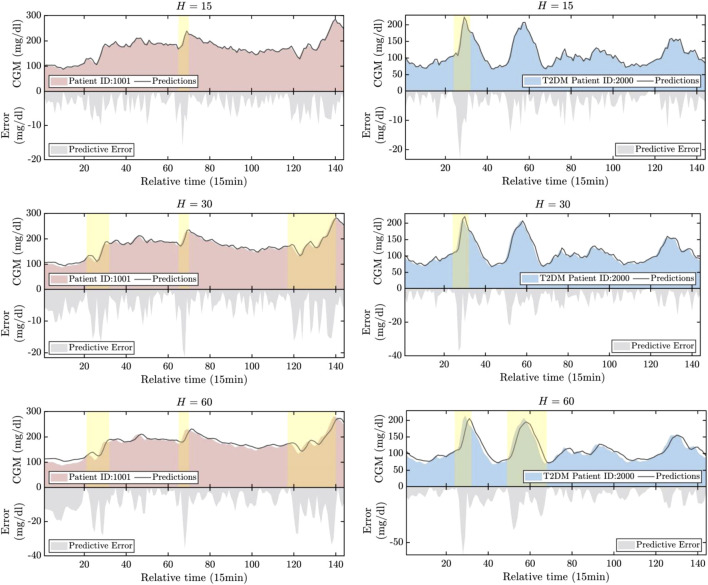
The visualization of the real values versus the predicted values.

The prediction results are illustrated in Figure 5. For illustrative purposes, we have selected the first patient samples from both the T1DM and T2DM datasets. Periods characterized by significant predictive errors are highlighted in the figure. Based on these illustrations, several key observations can be summarized as follows: 1) The progression of CGM for both T1DM and T2DM patients can be effectively tracked across all three prediction horizons. This indicates the promising accuracy of the proposed HETER in multi-patient diabetic blood glucose prediction. 2) The proposed HETER exhibits robust performance during relatively stable phases for both types of diabetic patients. This can be attributed to HETER’s ability to fuse both global and local continuous temporal information via its graph learning module and temporal enhancement module. 3) The predictive error significantly increases with the extension of prediction horizons, particularly during highlighted periods. This phenomenon highlights the challenge of capturing the temporal dynamics associated with sharp increases and decreases. The proposed method succeeds in capturing as much of the trend of short-term fluctuations as possible, particularly under shorter prediction horizons. However, there remains room for improvement during periods of intense fluctuations in longer prediction horizons.

**FIGURE 6:**
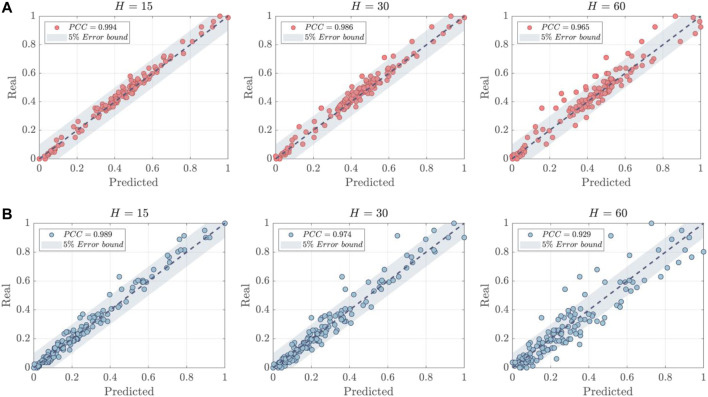
The correlation analysis of predictions. **(A)** T1DM Patient ID:1001. **(B)** T2DM Patient ID:2000.

The correlation analysis of the normalized predictive results is illustrated in Figure 6. The Pearson correlation coefficients (*PCC*) between actual values and predictions have also been calculated and are represented in Figure 6. Several findings were made as follows: 1) The stationarity of the data was found to be significantly different between T1DM and T2DM patients as demonstrated in the example. T1DM shows a more stable trend with the majority of data points situated in the middle range, approximating a normal distribution Eizirik et al. (2020). In contrast, the distribution of data points for T2DM patients demonstrates a significant drift, indicative of more severe fluctuations present in T2DM samples. This adds to the complexity and poses greater challenges for BGP models. 2) The proposed model exhibits strong generalization capabilities for both types of diabetic patients. While the instability of T2DM data may reduce the PCC values of the predictions, the majority of prediction errors are still confined within a 5% error bound, particularly when the prediction horizon *H* is small (15 or 30).

### 3.4 Parameter sensitivity analysis

**FIGURE 7:**
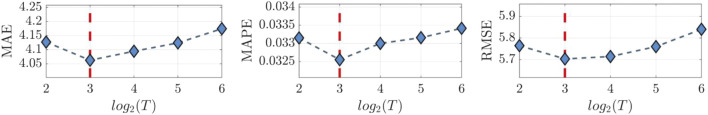
The sensitivity analysis of window size *T*.

**FIGURE 8:**
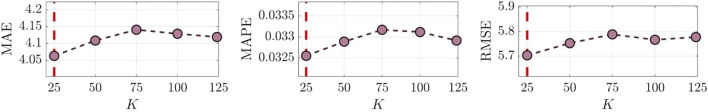
The sensitivity analysis of the hyperparameter *K*.

The impact of window sizes *T* and *K* are evaluated through parameter sensitivity analysis. The optimal results are highlighted by the red dashed lines. In this analysis, the prediction horizon *H* is fixed at 15. The *T* is varied from 4 to 64, and *K* is increased from 25 to 124, and their results are shown in Figures 7, 8, respectively. As depicted in Figure 7, HETER obtains optimal performance when the window size is set to 8. Beyond this point, the performance in terms of all three metrics (MAE, MAPE, and RMSE) shows a nearly linear proportionality to the window size, with performance decreasing as window size increases. This underscores the importance of selecting an appropriate window size. A smaller window size may not provide sufficient temporal information, while an excessively large window size might introduce unnecessary pattern associations and fluctuations, inevitably impacting the prediction accuracy adversely.

From Figure 8, the highest model accuracy is achieved when *K* is set to 25. According to Eq. 6, a larger *K* indicates denser adjacency information. However, an overly extensive relationship association might make it challenging for the prediction model to capture vital information, thus limiting prediction accuracy. These results also further verify the influence of graph relationships among multiple samples on prediction accuracy.

## 4 Conclusion

Inherent heterogeneity and uncertainty in multiple CGM datasets present significant constraints to the applicability of traditional personalized BGP models in multi-patient scenarios. In this study, a novel HETER model is proposed for the simultaneous prediction of blood glucose levels in multiple diabetic patients. First, the SSR method is utilized to align patient samples drawn from heterogeneous time series. Subsequently, multiple CGM datasets are structured as a graph, employing a graph structure learning module to capture global temporal information. To improve the model’s learning capability for continuous temporal characteristics, we incorporated a representation enhancement module into HETER, which allows it to highlight key information and further extract temporal representations. Additionally, we considered linear representations to enhance the model’s predictive stability. Finally, We conducted comprehensive experiments to evaluate our proposed HETER model against five comparable methods. The results of these experiments verify the superiority of our proposed SSR method and HETER model. The rising demand for effective and accurate tracking of the progression of multiple diabetic patients in clinical scenarios heightens the necessity to enhance existing methodologies. HETER is an important methodological advancement for predicting heterogeneous multi-patient CGM data using graph neural networks.

There are several potential directions for future research. First, we aim to further optimize the alignment process for heterogeneous CGM data. The generative diffusion model could be employed to align heterogeneous data by generating new data similar to historical observations. Second, we plan to incorporate additional related factors into the multi-patient BGP, including patient behavior, heart rate, and food intake. This could enhance the robustness and generalizability of the prediction model, while also improving the interpretability of the results. Third, knowledge distillation and dynamic graph convolution could be utilized to design a lightweight graph architecture. This would potentially reduce the storage and computational requirements of the model.

## Data Availability

The original contributions presented in the study are included in the article/Supplementary Material, further inquiries can be directed to the corresponding author.
