# Supplementary material for: Heterogeneous temporal representation for diabetic blood glucose prediction
Source: Front Physiol. 2023 Jul 17;14:1225638. doi: 10.3389/fphys.2023.1225638 (PMC10393041; doi:10.3389/fphys.2023.1225638)
Supplement: Supplementary file 1 [file DataSheet1.PDF]

Supplementary Material

1 SUPPLEMENTARY TABLES

Table S1. Symbols and semantics.

Symbol	Semantic
p	The dropout probability
t	The t-th data point of time series
n	The n-th sample of input data
s	The size of predicted step
H	The prediction horizon
T	The window size
B	The batch size of a data tensor
N	The number of samples
K	The number of selected relationships in graph construction.
$\boldsymbol{\mathcal{D}}$	The dataset
D	The time series in the dataset
d	The element in the time series
$\boldsymbol{X}$	The model input data
$\boldsymbol{Y}$	The predictive target
$\boldsymbol{A}$	Adjacent matrix
$\boldsymbol{W}$	The weighting matrix
$\boldsymbol{b}$	The bias term
$\boldsymbol{\mathcal{R}}$	The obtained representation
$[;]$	The concatenation operation

Table S2. Hyper-parameter settings.

Model	Parameter	Option range
Vanilla LSTM	Hidden size	$\{2^4, 2^5, 2^6\}$
Bi-LSTM	LSTM layers	1-3 (1 per step)
TPA-LSTM	Kernel size	3-9 (2 per step)
	CNN out channels	$\{2^2, 2^3, 2^4, 2^5, 2^6\}$
LSTNet	GRU hidden size	$\{2^4, 2^5, 2^6\}$
LSTNet	Skip window size	1-3 (1 per step)
	Skip GRU hidden size	$\{2^4, 2^5, 2^6\}$
Transformer	Encoder hidden size	$\{2^3, 2^4, 2^5, 2^6\}$
	Decoder hidden size	$\{2^3, 2^4, 2^5, 2^6\}$
	Encoder and decoder layers	$\{1, 2, 3\}$
	The numbers of heads	$\{2^2, 2^3, 2^4, 2^5\}$
	Embedding hidden size	100-300 (50 per step)
	The dimension of the model	$\{2^3, 2^4, 2^5, 2^6\}$
HETER	GCN hidden size	$\{2^4, 2^5, 2^6, 2^6\}$
	GCN out channel	$\{2^5, 2^6, 2^7\}$
	CNN kernel size	1-5 (2 per step)
	CNN out channel	10-50 (10 per step)
	GRU hidden size	$\{2^3, 2^4, 2^5, 2^6\}$
	GRU layers	1-3 (1 per step)

Table S3. Corresponding meanings and categories of acronyms.

Abbreviation	Meaning
BGP	Blood glucose prediction
CGM	Continuous glucose monitoring
DTW	Dynamic time warping
GCNs	Graph convolution networks
GRU	Gated recurrent unit
HTS	Heterogeneous time series
LSTM	Long short-term memory network
PAD	Padding
MLP	Multilayer perceptron network
MAE	Mean absolute error
MAPE	Mean absolute percentage error
MSE	Mean squared error
RMSE	Root mean square error
RF	Random forester
ReLU	Rectified linear unit
SVM	Support vector machine
T1DM	Type 1 diabetes mellitus
T2DM	Type 2 diabetes mellitus
TRA	Truncation
TA	Temporal attention
